# Horizontal transfer and proliferation of Tsu4 in Saccharomyces paradoxus

**DOI:** 10.1186/s13100-018-0122-7

**Published:** 2018-06-12

**Authors:** Casey M. Bergman

**Affiliations:** Department of Genetics and Institute of Bioinformatics, University of Georgia, East Green St., Athens, GA, 30602 USA

**Keywords:** Transposable element, Horizontal transfer, Yeast, Genome rearrangement

## Abstract

**Background:**

Recent evidence suggests that horizontal transfer plays a significant role in the evolution of of transposable elements (TEs) in eukaryotes. Many cases of horizontal TE transfer (HTT) been reported in animals and plants, however surprisingly few examples of HTT have been reported in fungi.

**Findings:**

Here I report evidence for a novel HTT event in fungi involving *Tsu4* in *Saccharomyces paradoxus* based on (i) unexpectedly high similarity between *Tsu4* elements in *S. paradoxus* and *S. uvarum*, (ii) a patchy distribution of *Tsu4* in *S. paradoxus* and general absence from its sister species *S. cerevisiae*, and (iii) discordance between the phylogenetic history of *Tsu4* sequences and species in the *Saccharomyces sensu stricto* group. Available data suggests the HTT event likely occurred somewhere in the Nearctic, Neotropic or Indo-Australian part of the *S. paradoxus* species range, and that a lineage related to *S. uvarum* or *S. eubayanus* was the likely donor species. The HTT event has led to massive proliferation of *Tsu4* in the South American lineage of *S. paradoxus*, which exhibits partial reproductive isolation with other strains of this species because of multiple reciprocal translocations. Full-length *Tsu4* elements are associated with both breakpoints of one of these reciprocal translocations.

**Conclusions:**

This work shows that comprehensive analysis of TE sequences in essentially-complete genome assemblies derived from long-read sequencing provides new opportunities to detect HTT events in fungi and other organisms. This work also provides support for the hypothesis that HTT and subsequent TE proliferation can induce genome rearrangements that contribute to post-zygotic isolation in yeast.

**Electronic supplementary material:**

The online version of this article (10.1186/s13100-018-0122-7) contains supplementary material, which is available to authorized users.

## Main Text

Horizontal transfer is increasingly thought to play an important role in shaping the diversity of transposable elements (TEs) in eukaryotic genomes [1–3]. Since the initial discovery of horizontal transfer of the *P* element from *Drosophila willistoni* to *D. melanogaster* [4], a large number of cases of horizontal TE transfer (HTT) have been reported, especially among animals species (data compiled in [5]). However, surprisingly few cases of HTT have been reported in fungi [6–12], despite an abundance of genomic resources in this taxonomic group. Advances in long-read whole genome shotgun sequencing now allow comprehensive analysis of TE sequences in high-quality genome assemblies, and may therefore provide new opportunities for detecting HTT events in fungi and other organisms.

For example, a recent study by Yue et al. [13] reported essentially-complete PacBio genome assemblies for seven strains of *S. cerevisiae* and five strains of *S. paradoxus*. Analysis of TEs in these assemblies revealed a surprisingly high copy number for the *Ty4* family in one strain of *S. paradoxus* from South America (UFRJ50816; *n* =23 copies) [13]. This was a noteworthy observation for two reasons: (i) *Ty4* is typically found at low copy number in yeast strains [14–16], and (ii) S. American strains of *S. paradoxus* exhibit partial reproductive isolation with other strains of this species, which principally results from multiple reciprocal translocations thought to have arisen by unequal crossing-over between dispersed repetitive elements such as *Ty* elements [17].

I independently replicated the curious observation of exceptionally high *Ty4* copy number in *S. paradoxus* UFRJ50816 using a RepeatMasker-based annotation pipeline similar to that described in [11], which identifies and classifies *Ty* elements as full-length, truncated, or solo long terminal repeats (LTRs). Using the results of this initial annotation, I generated a multiple alignment of all full-length *Ty4* elements identified in these 12 assemblies. Preliminary phylogenetic analysis revealed that the full-length *Ty4* elements from *S. paradoxus* UFRJ50816 formed a monophyletic clade of very similar sequences that were highly divergent from other full-length *Ty4* elements identified in *S. cerevisiae* (S288c, Y12, YPS128) or *S. paradoxus* (N44). Surprisingly, BLAST analysis at NCBI using representative members of this divergent *Ty4*-like clade revealed that they were more similar to the *Tsu4* element from the related yeast species *S. uvarum* (Genbank: AJ439550) [18] than they were to the original *S. cerevisiaeTy4* query sequence (Genbank: S50671). This result suggested that the unusually high copy number of *Ty4* in *S. paradoxus* UFRJ50816 reported by Yue et al. [13] could actually be the consequence of rapid expansion of *Tsu4* following a HTT event from a *S. uvarum*-like donor.

To better characterize *Ty4* and *Tsu4* content in *S. cerevisiae* and *S. paradoxus*, I first identified a canonical *S. paradoxusTsu4* element. To do this, I included the *S. uvarumTsu4* query sequence in the TE library from [11] and re-annotated *Ty* elements in *S. paradoxus* UFRJ50816 using the same RepeatMasker-based strategy as above. I also performed *de novo* identification of full-length LTR elements in *S. paradoxus* UFRJ50816 using LTRharvest [19], then overlapped results from RepeatMasker and LTRharvest to identify full-length *Tsu4* elements, generated a consensus sequence from these elements, and finally identified the genomic copy (chrII:554570-560566) that clustered most closely with the consensus sequence of full-length *Tsu4* elements in a neighbor-joining tree. The canonical *S. paradoxusTsu4* element is 5,997 bp in length, with 293 bp LTRs and two overlapping reading frames predicted to encode GAG (358 amino acids) and POL (1419 amino acids). Codon-based alignment with PRANK [20] and estimation of dN/dS ratios under PAML model M0 using ETE [21, 22] revealed that selective constraints have operated on both GAG (0.34) and POL (0.21) sequence divergence between *S. uvarum* and *S. paradoxusTsu4* canonical elements. Estimates of dS for GAG (0.23) and POL (0.24) between *S. uvarum* and *S. paradoxusTsu4* canonical elements were over four times lower than the median dS (1.01) for nuclear coding sequences estimated under M0 for these species using 4554 high-confidence alignments from [23]. This observation supports HTT of *Tsu4* and argues against various modes of vertical inheritance to explain the similarity between *S. paradoxus* and *S. uvarumTsu4*.

I then performed a final annotation of *Ty* elements in all 12 assemblies from Yue et al. [13] using the TE library from [11] plus the newly-identified *S. paradoxusTsu4* canonical element. Full-length, truncated and solo LTR counts for *Tsu4* and *Ty4* can be found in Table 1. Similar data for all *Ty* families in these genomes can be found in Additional file 1 and coordinates of all annotated *Ty* elements in these genomes can be found in Additional file 2. This improved annotation revealed a low copy number of full-length *Ty4* elements for *S. cerevisiae* S288c, Y12, and YPS128 as well as *S. paradoxus* N44, which is typical of this family [14–16]. Solo LTRs for *Ty4* were found in all strains of *S. cerevisiae* and *S. paradoxus* with PacBio data from from Yue et al. [13]. Solo LTRs arise by intra-element LTR-LTR recombination and serve as useful markers of past transpositional activity [24]. These results suggest that *Ty4* was present in the common ancestor of both *S. cerevisiae* and *S. paradoxus* and that this family has been maintained at low copy number or become inactive in different lineages of each species.

**Table 1:** *Ty4* and *Tsu4* content in *S. cerevisiae* and *S. paradoxus* PacBio assemblies from Yue et al. [13]

Species	Strain	# Ty4 full	# Ty4 truncated	# Ty4 solo LTR	# Tsu4 full	# Tsu4 truncated	# Tsu4 solo LTR
Scer	S288c	3	0	14	0	0	0
Scer	DBVPG6044	0	0	18	0	0	0
Scer	DBVPG6765	0	0	5	0	0	0
Scer	SK1	0	0	14	0	0	0
Scer	Y12	4	1	14	0	0	0
Scer	YPS128	4	0	13	0	0	0
Scer	UWOPS03-461.4	0	0	60	0	0	0
Spar	CBS432	0	2	46	0	0	0
Spar	N44	3	3	106	0	0	0
Spar	YPS138	0	0	49	1	0	18
Spar	UFRJ50816	0	1	45	22	2	105
Spar	UWOPS91-917.1	0	0	40	1	1	88

Numbers of full-length elements (both LTRs present and internal region present with > 95% coverage of canonical internal region), truncated elements (internal region present with < 95% coverage of canonical internal region), or solo LTRs (no match to internal region) were estimated using a RepeatMasker (version 4.0.5) based strategy and a custom library of *Ty* elements from [11] supplemented with *S. paradoxusTsu4*

In contrast, I found evidence for full-length *Tsu4* sequences in only three strains of *S. paradoxus* from S. America (UFRJ50816, *n*=22), N. America (YPS138, *n*=1) and Hawaii (UWOPS91-917.1, *n*=1) (Table 1). S. American, N. American, and Hawaiian lineages of *S. paradoxus* form a monophyletic group that is distinct from a clade containing European and Far-Eastern lineages from the Old World [13, 25]. *S. paradoxus* strains with full-length copies of *Tsu4* were devoid of full-length *Ty4* elements, and *vice versa*. Crucially, only these three *S. paradoxus* strains had solo LTRs for *Tsu4*, suggesting they are the only lineages in which *Tsu4* has been active in the past. Consistent with a more recent presence in the *S. paradoxus* genome, solo LTRs for *Tsu4* exhibited ≥3-fold lower average pairwise divergence than *Ty4* solo LTRs relative to their respective canonical elements within the genomes of UFRJ50816 (*Tsu4*: 0.02, *Ty4*: 0.10), YPS138 (*Tsu4*: 0.03, *Ty4*: 0.10) and UWOPS91-917.1 (*Tsu4*: 0.04, *Ty4*: 0.12).

The 22 full-length copies of *Tsu4* identified in UFRJ50816 are distributed on 11 different chromosomes, indicating proliferation in UFRJ50816 is not simply due to tandem duplication. Twelve of the full-length *Tsu4* copies in UFRJ50816 are flanked by 5 bp target site duplications (TSDs) that can be automatically identified by LTRharvest [19] and 21 are within ± 1 kb of a tRNA gene, similar to what has been reported previously for *Ty4* in *S. cerevisiae* [15, 26]. Diagnostic hallmarks of active *Ty* element transposition such as 5 bp TSDs and tRNA targeting suggest that *Tsu4* has been recently active in UFRJ50816 and also argue against the possibility that *Tsu4* sequences in *S. paradoxus* are due to contamination in DNA samples.

I next confirmed the general absence of *Tsu4* in *S. cerevisiae* by BLAST analysis of an additional 336 *S. cerevisiae* whole genome shotgun (WGS) assemblies at NCBI (taxid: 4932), which revealed only one nearly complete sequence with high similarity to *Tsu4* from a *S. cerevisiae* strain isolated from a rum distillery in the West Indies (> 80% coverage and > 80% identity, see below) [27]. The patchy distribution of *Tsu4* sequences in *S. paradoxus* and general absence from *S. cerevisiae* suggests that this element was not present in the common ancestor of these species, and instead was recently acquired by a *S. paradoxus* lineage somewhere in the Nearctic, Neotropic or Indo-Australian region, possibly in a strain lacking an active *Ty4*. In principle, the patchy distribution of *Tsu4* in *S. paradoxus* and absence from *S. cerevisiae* could be explained by vertical inheritance from the common ancestor of *S. uvarum*, *S. cerevisiae* and *S. paradoxus* followed by multiple losses in *S. cerevisiae* and *S. paradoxus*, however the much lower dS between *S. uvarum* and *S. paradoxusTsu4* coding regions relative to host genes argues against this scenario.

To provide further support for the hypothesis that *Tsu4* recently invaded *S. paradoxus* by HTT, I constructed a maximum likelihood phylogeny of all full-length *Ty4* and *Tsu4* sequences identified in the 12 strains of *S. cerevisiae* and *S. paradoxus* from Yue et al. [13] using RAxML [28]. In this analysis, I also included all complete or nearly-complete *Tsu4* elements identified by BLAST in 392 *Saccharomyces* WGS assemblies at NCBI (taxid: 4930) that had high similarity to the *S. uvarumTsu4* query sequence (> 80% coverage and > 80% identity). These additional 12 *Tsu4* sequences include three sequences from the same strain of *S. uvarum* in which *Tsu4* was discovered, one sequence from *S. mikatae*, one sequence from *S. kudriavzevii*, one sequence each from four strains of *S. pastorianus*, two sequences from an unknown *Saccharomyces* species (strain M14) isolated in China that is involved in lager brewing, and the single sequence from *S. cerevisiae* (strain 245) mentioned above (Additional file 3) [27, 29–31]. *S. mikatae* is the most closely related outgroup species to the *S. cerevisiae*/*S. paradoxus* clade, followed by *S. kudriavzevii*, then a clade containing *S. uvarum* and *S. eubayanus* (reviewed in [32]). *S. pastorianus* is a hybrid species used in lager brewing containing subgenomes from *S. cerevisiae* and *S. eubayanus* [30, 33, 34]. The multiple sequence alignment and maximum likelihood tree for this dataset can be found in Additional files 4 and 5, respectively.

**Fig. 1:**
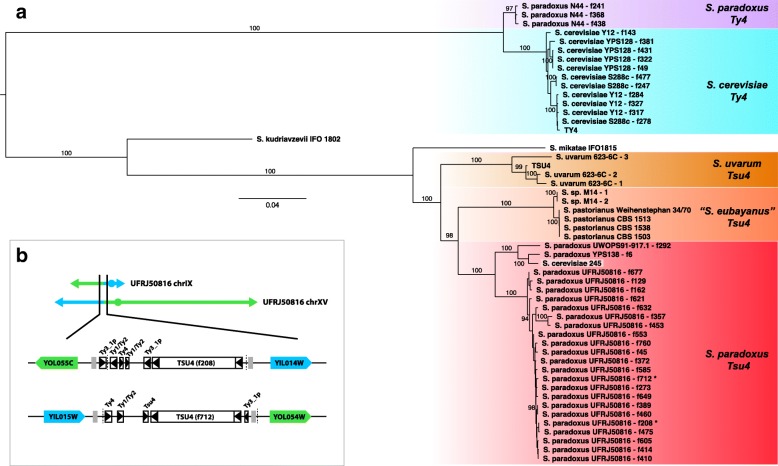
Evolution of the *Ty4*/*Tsu4* super-family in the *Saccharomyces sensu stricto* species group. **a**. Maximum likelihood phylogeny of all full-length *Ty4* and *Tsu4* elements from 12 strains of *S. cerevisiae* and *S. paradoxus* with PacBio assemblies from Yue et al. [13] plus all complete or nearly-complete *Tsu4* elements identified in *Saccharomyces* WGS assemblies at NCBI. Labels are shown for branches in the maximum likelihood tree that are supported by ≥ 90% of bootstrap replicates. The scale bar for branch lengths is in substitutions per site, and the tree is midpoint rooted. *Tsu4* sequences from hybrid strains (*S. pastorianus* and *Saccharomyces sp. M14*) were assigned to the *S. eubayanus* subgenome and presumed to have originated in *S. eubayanus*. **b**. Genome organization of the reciprocal translocation between chrIX and chrXV in UFRJ50816. Sequences from the standard arrangement chrIX are shown in blue, and sequences from the standard arrangement chrXV are shown in green. Protein-coding genes, tRNA genes, and solo LTRs are shown approximately to scale as solid arrows, grey rectangles and boxed arrowheads, respectively. Approximate translocation breakpoints in UFRJ50816 based on whole genome alignments can be localized to chrIX:252268-259232 and chrXV:320536-328356 (dashed lines). Full-length *Tsu4* elements are present in both translocation breakpoints. The *Tsu4* elements associated in the chrIX and chrXV reciprocal translocation between are denoted by asterices in panel **a**

Figure 1a clearly shows that *Tsu4*-like sequences form a well-supported monophyletic clade that is distinct from the *Ty4* lineage present in *S. cerevisiae* and *S. paradoxus*. All *S. paradoxusTsu4* sequences form a single clade that also contains the *Tsu4* sequence from *S. cerevisiae* strain 245, suggesting one initial HTT event into *S. paradoxus* followed by a secondary HTT event from *S. paradoxus* into *S. cerevisiae*. The most closely-related lineage to the *S. paradoxusTsu4* clade is a clade containing sequences from *S. pastorianus* and *Saccharomyces sp. M14*, followed by a clade containing sequences from *S. uvarum*. The grouping of *Tsu4* sequences from the hybrid species *S. pastorianus* with those from *S. paradoxus* and *S. uvarum* can most parsimoniously be explained if *Tsu4* sequences from *S. pastorianus* are derived from the *S. eubayanus* component of the hybrid genome (see below). If this is true, then *Tsu4* in *S. paradoxus* could plausibly have arisen *via* HTT from *S. eubayanus*, the sister species to *S. uvarum*. *Tsu4* sequences from both *S. mikatae* and *S. kudriavzevii* are outgroups to the crown *Tsu4* lineage, but group more closely to the *Tsu4* lineage than to the *Ty4* lineage with strong support. The observation that *S. mikatae* groups more closely *Tsu4* with the crown *Tsu4* lineage than *S. kudriavzevii* is incompatible with the accepted species tree [32], suggesting unequal rates of evolution or another potential HTT event involving *Tsu4* between *S. mikatae* and the ancestor of *S. uvarum* and *S. eubayanus*. Despite unresolved issues with some aspects of the current *Tsu4* phylogeny, the fact that *S. uvarum* is the closest pure species clustering with the *S. paradoxus* clade is clearly incompatible with the accepted tree for these species [32] and this discordance provides support for the conclusion that *Tsu4* arose in *S. paradoxus* by HTT from *S. uvarum* or a closely related species like *S. eubayanus*.

To address the origin of *Tsu4* sequences in *S. pastorianus* genomes and better understand potential donors for the HTT event, I aligned *S. pastorianus* WGS assemblies to a pan-genome comprised of *S. cerevisiae* S288c [13] and *S. eubayanus* FM1318 [34], a monosporic derivative of the *S. eubayanus* type strain which was isolated from Northwestern Patagonia, Argentina [33]. The *Tsu4* sequences from all four strains of *S. pastorianus* are contained on scaffolds that align best to scaffolds from the *S. eubayanus* subgenome (Additional file 6), consistent with the general lack of *Tsu4* in *S. cerevisiae* and the phylogenetic clustering of *S. pastorianusTsu4* sequences with those from *S. uvarum*. In fact, *Tsu4* sequences for all four *S. pastorianus* strains align to the same location in *S. eubayanus* (scaffold NC_030972.1), which together with their tight clustering on the tree suggests they are alleles of the same insertion event. Likewise, alignment of the *Saccharomyces sp. M14* genome to a pan-genome of *S. eubayanus* and *S. cerevisiae* revealed large scaffolds that aligned to either scaffolds from *S. eubayanus* or chromosomes from *S. cerevisiae* (Additional file 7), in a similar pattern to the *bona fideS. pastorianus* group 2/Frohberg strain W34/70 (Additional file 8). Thus, *Saccharomyces sp. M14* appears to be a hybrid of *S. cerevisiae* and *S. eubayanus* and may possibly be a previously-unidentified strain of *S. pastorianus*. The two *Tsu4* sequences from *Saccharomyces sp. M14* align to a different *S. eubayanus* scaffold (NC_030977.1) and form a cluster on the tree that is distinct from the *S. pastorianusTsu4* sequences, suggesting they arose from different transposition events. None of the *S. pastorianus*/*Saccharomyces sp. M14Tsu4* insertions are found in the *S. eubayanus* reference genome, nor are any other complete or nearly-complete *Tsu4* sequences. Several distinct clades have been identified in *S. eubayanus* using whole genome sequence data: the reference strain (FM1318) is found in the Patagonia B-1 clade, while North American, Tibetan and the *S. eubayanus* subgenomes of *S. pastorianus* strains are found in the related but distinct Holoarctic clade [35]. Overall, the *S. eubayanus* subgenome localization and close affinity of *S. pastorianus*/*Saccharomyces sp. M14Tsu4* sequences with *S. paradoxusTsu4* sequences suggest that *S. eubayanus* is a viable donor for the *Tsu4* element that invaded *S. paradoxus*, and that *Tsu4* has been recently active in the Holoarctic clade of *S. eubayanus*.

Within *S. paradoxus*, the deepest well-supported branches in the *S. paradoxusTsu4* clade are between N. American/Hawaiian and S. American *S. paradoxus* lineages, suggesting the HTT event predates separation of these lineages and could therefore have occurred anywhere in the ancestral range for *S. paradoxus* in the Nearctic, Neotropic or Indo-Australian regions. All of the *Tsu4* sequences in UFRJ50816 form a single clade, but bootstrap support for most branches within this clade are low, consistent with a recent proliferation event occurring after separation of S. American *S. paradoxus* from N. American and Hawaiian lineages. The single *S. cerevisiaeTsu4* sequence found in a strain 245 (isolated from the French West Indies) clusters strongly with the *Tsu4* sequence from the N. American *S. paradoxus* strain YPS138. Introgression of *S. paradoxus* DNA into *S. cerevisiae* has been observed previously [36–41], and thus introgression from a N. American-like lineage of *S. paradoxusTsu4* into *S. cerevisiae* in the Caribbean could explain this secondary HTT event. As in N. American and Hawaiian lineages of *S. paradoxus*, *Tsu4* in this *S. cerevisiae* lineage has not led to widespread proliferation, suggesting the high copy number of *Tsu4* in UFRJ50816 is exceptional.

*S. paradoxus* UFRJ50816 was originally thought to represent a distinct species called *S. cariocanus* based on partial reproductive isolation with *S. paradoxus* tester strains [36, 42, 43]. Five reciprocal translocations have been identified on the lineage leading to UFRJ50816 relative to the standard *S. paradoxus* karyotype that account for most of this reproductive isolation [13, 17, 44]. To test whether the recent proliferation of of *Tsu4* in UFRJ50816 has induced genome rearrangements involved in reproductive isolation, I identified translocation breakpoints in *S. paradoxus* UFRJ50816 relative to the standard karyotype *S. paradoxus* strain CBS432 using Mummer [45] and Ribbon [46]. Only one out of five translocations showed clear evidence for *Tsu4* sequences at both breakpoints. Intriguingly, both breakpoints of the translocation between chrIX and chrXV (which has recently been shown to reduce spore viability by approximately 50% [44]) each contained a full-length *Tsu4* element (Fig. 1b). These two elements are from the major clade of *Tsu4* sequences found only in UFRJ50816, and are oriented in the directions expected if they were involved in a reciprocal exchange event. These results indicate that recent ectopic exchange among *Tsu4* sequences is not the primary cause of the majority of translocations in UFRJ50816, however *Tsu4* proliferation may have facilitated some genome rearrangements in the UFRJ50816 lineage.

In conclusion, here I report evidence for a novel HTT event in fungi involving *Tsu4* in *S. paradoxus* based on (i) unexpectedly high similarity between *Tsu4* elements in *S. paradoxus* and *S. uvarum*, (ii) a patchy distribution of *Tsu4* in *S. paradoxus* and general absence from its sister species *S. cerevisiae*, and (iii) discordance between the phylogenetic history of *Tsu4* sequences and host species trees. Based on available data, the most parsimonious scenario for the evolution of the *Ty4*/*Tsu4* super-family is that an ancestral *Ty4*/*Tsu4*-like element was present in the ancestor of the *Saccharomyces sensu stricto* group, which diversified into *Ty4* along the lineage leading to *S. cerevisiae*/*S. paradoxus* and into *Tsu4* on the lineage leading to *S. uvarum*/*S. eubayanus*. Subsequently, a HTT event occurred introducing *Tsu4* into the ancestor of non-Old World *S. paradoxus*, most likely from *S. uvarum*, *S. eubayanus* or a related species. This scenario is plausible since both *S. uvarum* and *S. eubayanus* have been sampled from sites in N. America and S. America that overlap or are in close proximity to the predicted range of *S. paradoxus* [33, 35, 47–49], and *S. uvarum* has been isolated from the same field sites as *S. paradoxus* in N. America [50]. Other more complex scenarios are also possible but would involve additional HTT events, such as HTT from *S. uvarum*/*S. eubayanus* to *S. paradoxusvia* intermediate species or multiple HTT events from an unidentified species into the ancestor of non-Old World *S. paradoxus* and *S. uvarum*, *S. eubayanus* or their common ancestor. A number of open questions remain about the *Tsu4* HTT event, including which lineage donated *Tsu4* to *S. paradoxus*, where the HTT event occurred, how widely *Tsu4* has spread in *S. paradoxus*, and whether the HTT event was mediated by interspecific hybridization or some other mechanism. I also show that full-length *Tsu4* elements are associated with the breakpoints of a reciprocal translocation that provides partial reproductive isolation between lineages of *S. paradoxus* from S. America and the rest of the world. These findings together with related work on *Ty2* in *S. cerevisiae* [8, 11, 51] provide support for the hypothesis that HTT and subsequent proliferation can induce genome rearrangements that contribute to post-zygotic isolation in yeast.

## Additional files

Additional file 1 *Ty* content in *S. cerevisiae* and *S. paradoxus* PacBio assemblies. Numbers of full-length elements (both LTRs and internal region present with > 95% coverage of canonical internal region), truncated elements (internal region present with < 95% coverage of canonical internal region), or solo LTRs (no match to internal region) were identified using a RepeatMasker (version 4.0.5) based strategy and a custom library of *Ty* elements from [11] supplemented with *S. paradoxusTsu4*. (TSV 1.20 kb)

Additional file 2 Coordinates of *Ty* elements in *S. cerevisiae* and *S. paradoxus* PacBio assemblies. Zip file of BED-formatted genome annotations for 12 strains of *S. cerevisiae* and *S. paradoxus* with PacBio assemblies. Full-length elements (f), truncated elements (t), or solo LTRs (s) were identified using a RepeatMasker (version 4.0.5) based strategy and a custom library of *Ty* elements from [11] supplemented with *S. paradoxusTsu4*. (ZIP 91.1 kb)

Additional file 3 Summary of complete or nearly-complete *Tsu4* sequences identified in *Saccharomyces* whole genome assemblies at NCBI. Accession number and coordinates of 12 complete or nearly-complete *Tsu4* elements identified by BLAST in 392 *Saccharomyces* (taxid: 4930) WGS assemblies at NCBI that had high similarity (> 80% coverage and > 80% identity) to the *Tsu4* query sequence (Genbank: AJ439550). (CSV 1.77 kb)

Additional file 4 Multiple sequence alignment of *Ty4* and *Tsu4* elements in *Saccharomyces sensu stricto* species. Multiple sequence alignment of all full-length *Ty4*/*Tsu4* elements from 12 strains of *S. cerevisiae* and *S. paradoxus* with PacBio assemblies from Yue et al. [13] plus all complete or nearly-complete *Tsu4* elements identified in *Saccharomyces* WGS assemblies at NCBI (> 80% coverage and > 80% identity relative to the *Tsu4* query sequence from *S. uvarum*). Fasta files of *Ty4*/*Tsu4* sequences from all strains plus the *Ty4* and *Tsu4* query sequences were concatenated together and aligned using MAFFT (version 7.273-e; options: –thread 28) [52]. (TXT 335 kb)

Additional file 5 Maximum likelihood tree file for *Ty4* and *Tsu4* elements in *Saccharomyces sensu stricto* species. Newick-formatted file of the maximum-likelihood tree of all full-length *Ty4*/*Tsu4* elements from 16 strains of *S. cerevisiae* and *S. paradoxus* plus all complete or nearly-complete *Tsu4* elements identified in *Saccharomyces* WGS assemblies at NCBI. Maximum-likelihood phylogenetic analysis was performed on the multiple alignment in Additional file 4 using RAxML (version: 8.2.4; options -T 28 -f a -x 12345 -p 12345 -N 100 -m GTRGAMMA) [28] excluding positions 1-166 and 6086-6476. (TXT 3.79 kb)

Additional file 6 Coordinates of best-matches to scaffolds from *S. pastorianus* and *Saccharomyces sp. M14* containing *Tsu4* sequences *vs.* a pan-genome of *S. eubayanus* and *S. cerevisiae* genomes. *S. pastorianus* and *Saccharomyces sp. M14* scaffolds were aligned to a pan-genome composed of scaffolds from *S. eubayanus* FM1318 (Genbank: GCF_001298625.1) and chromosomes from *S. cerevisiae* S288c (from [13]). Alignments were generated using nucmer (default parameters), delta-filter (options: -1 -l 2000), and show-coords (options: -lTH) in mummer 3.23 [45]. (TSV 5.15 kb)

Additional file 7 Dot-plot of the Chinese lager strain *Saccharomyces sp. M14vs.* a pan-genome of *S. eubayanus* and *S. cerevisiae* genomes. Dot-plot of *Saccharomyces sp. M14* scaffolds aligned to a pan-genome composed of scaffolds from *S. eubayanus* FM1318 (Genbank: GCF_001298625.1) and chromosomes from *S. cerevisiae* S288c (from [13]), showing that *Saccharomyces sp. M14* contains subgenomes from both species and that this strain may be a previously-unidentified strain of the lager brewing species *S. pastorianus*. The dot-plot was generated using nucmer (default parameters) and mummerplot (options: --size large -fat --color -f --png) in mummer 3.23 [45]. (PDF 58.8 kb)

Additional file 8 Dot-plot of the *S. pastorianus* group 2/Frohberg strain W34/70 *vs.* a pan-genome of *S. eubayanus* and *S. cerevisiae* genomes. Dot-plot of *S. pastorianus* group 2/Frohberg strain W34/70 scaffolds aligned to a pan-genome composed of scaffolds from *S. eubayanus* (Genbank: GCF_001298625.1) and chromosomes from *S. cerevisiae* (S288c from [13]), showing that *S. pastorianus* group 2/Frohberg strain W34/70 contains subgenomes from both species in a similar pattern as for *Saccharomyces sp. M14* (see Additional file 7). The dot-plot was generated using nucmer (default parameters) and mummerplot (options: --size large -fat --color -f --png) in mummer 3.23 [45]. (PDF 69.8 kb)

## Abbreviations

HTT: Horizontal TE transfer
LTR: Long terminal repeat
TE: Transposable element
TSD: Target site duplication
