# Supplementary figures and images for: Horizontal transfer and proliferation of Tsu4 in Saccharomyces paradoxus
Source: Mob DNA. 2018 Jun 12;9:18. doi: 10.1186/s13100-018-0122-7 (PMC5998506; doi:10.1186/s13100-018-0122-7)

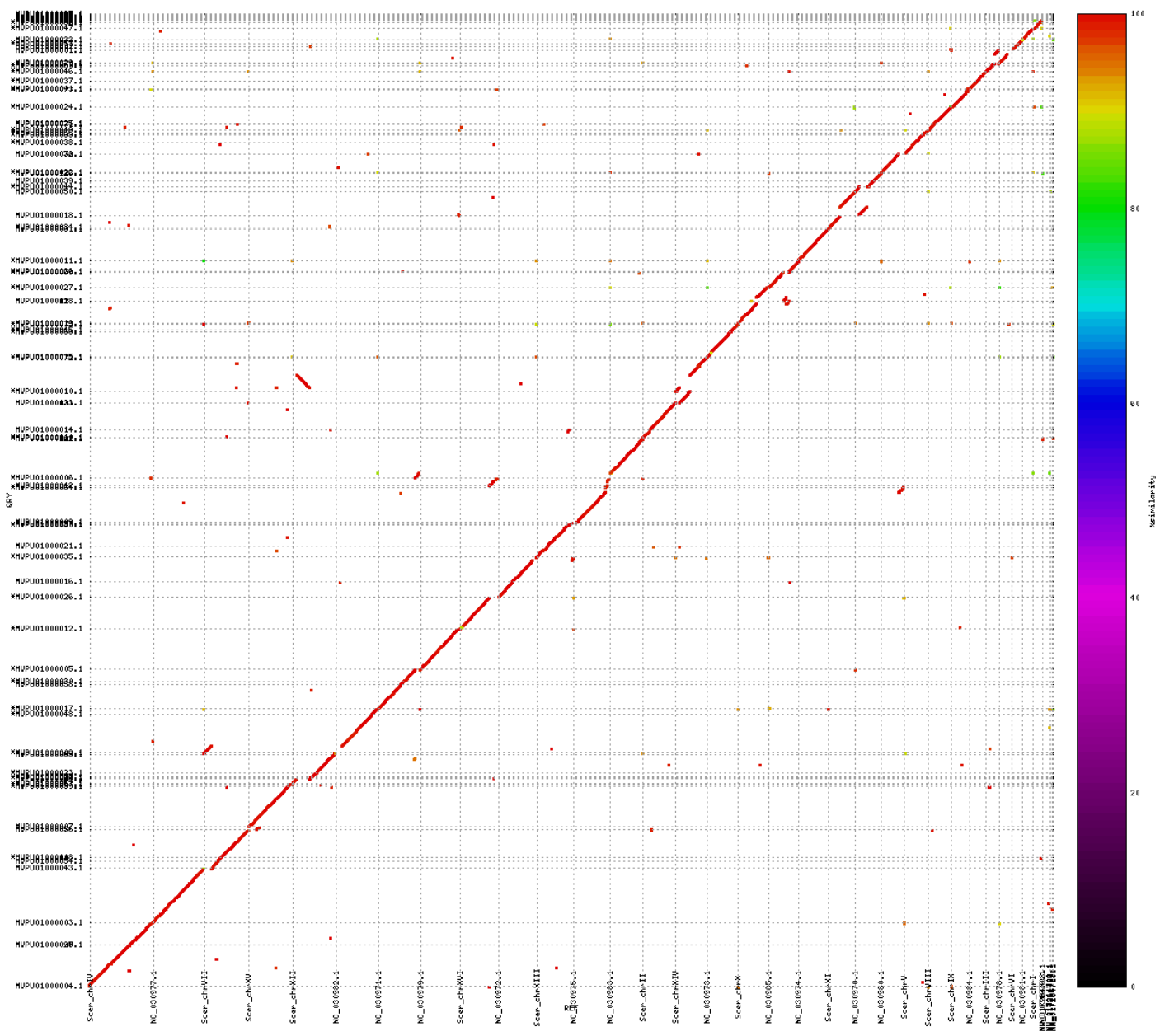

Supplement: Supplementary file 7 — Dot-plot of the Chinese lager strain Saccharomyces sp. M14vs. a pan-genome of S. eubayanus and S. cerevisiae genomes. Dot-plot of Saccharomyces sp. M14 scaffolds aligned to a pan-genome composed of scaffolds from S. eubayanus FM1318 (Genbank: GCF_001298625.1) and chromosomes from S. cerevisiae S288c (from [13]), showing that Saccharomyces sp. M14 contains subgenomes from both species and that this strain may be a previously-unidentified strain of the lager brewing species S. pastorianus. The dot-plot was generated using nucmer (default parameters) and mummerplot (options: --size large -fat --color -f --png) in mummer 3.23 [45]. (PDF 58.8 kb) [file 13100_2018_122_MOESM7_ESM.pdf]

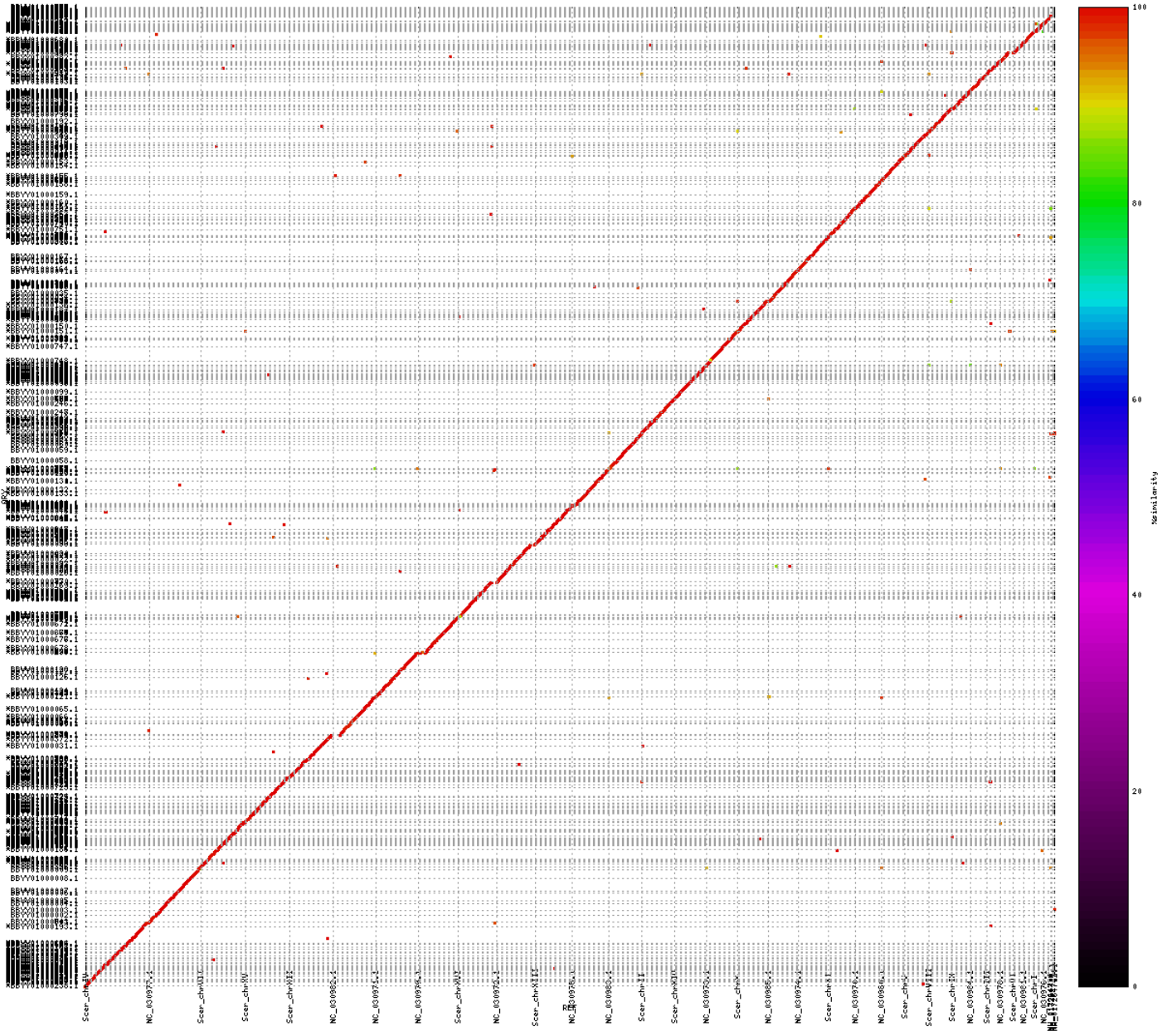

Supplement: Supplementary file 8 — Dot-plot of the S. pastorianus group 2/Frohberg strain W34/70 vs. a pan-genome of S. eubayanus and S. cerevisiae genomes. Dot-plot of S. pastorianus group 2/Frohberg strain W34/70 scaffolds aligned to a pan-genome composed of scaffolds from S. eubayanus (Genbank: GCF_001298625.1) and chromosomes from S. cerevisiae (S288c from [13]), showing that S. pastorianus group 2/Frohberg strain W34/70 contains subgenomes from both species in a similar pattern as for Saccharomyces sp. M14 (see Additional file 7). The dot-plot was generated using nucmer (default parameters) and mummerplot (options: --size large -fat --color -f --png) in mummer 3.23 [45]. (PDF 69.8 kb) [file 13100_2018_122_MOESM8_ESM.pdf]
